# Surface science studies of the coverage dependent adsorption of methyl acetate and methyl propanoate on graphite†

**DOI:** 10.1039/d4ra04466e

**Published:** 2024-11-05

**Authors:** Jack E. Fulker, Wendy A. Brown

**Affiliations:** a Department of Chemistry, University of Sussex Falmer Brighton BN1 9QJ UK w.a.brown@sussex.ac.uk

## Abstract

Complex organic molecules (COMs) have been detected in a wide range of astrophysical environments, including the interstellar medium, comets and proto-planetary disks. The icy mantles that form on dust grains in these environments are thought to be the chemical nurseries that allow the formation of many of the COMs that have been identified. As such, the adsorption, thermal processing and desorption of COMs from dust grain surfaces are important in understanding the astrochemical networks as a whole. To study these processes, surface science techniques (temperature programmed desorption (TPD) and reflection absorption infrared spectroscopy (RAIRS)) have been used to investigate ices of the simple esters, methyl acetate and methyl propanoate, adsorbed on a graphitic dust grain analogue surface (highly oriented pyrolytic graphite, HOPG) at 28 K. From the TPD experiments, kinetic parameters have been determined for the desorption of the esters from graphite. The data show a clear coverage dependence for the desorption energies and pre-exponential factors in the sub-monolayer regime. For methyl acetate, the desorption energies and pre-exponential factors range from 57.1 ± 0.4 to 47.2 ± 0.3 kJ mol^−1^ and 3.1 × 10^19±0.2^ to 1.6 × 10^19±0.1^ s^−1^ respectively. For methyl propanoate the same parameters range from 57.0 ± 0.1 to 51.0 ± 0.1 kJ mol^−1^ and 7.7 × 10^19±0.1^ to 4.4 × 10^19±0.1^ s^−1^. As expected, neither ester shows coverage dependent values for multilayer ices. The determined desorption energies and pre-exponential values for the multilayer ices are 43.5 ± 0.9 kJ mol^−1^ and 4.2 × 10^32±0.4^ molecules cm^−2^ s^−1^ for methyl acetate and 45.7 ± 0.9 kJ mol^− 1^ and 8.7 × 10^29±0.4^ molecules cm^−2^ s^−1^ for methyl propanoate. Experimental RAIRS data were also recorded, showing that the ices undergo an irreversible phase change from an amorphous to a crystalline structure when thermally processed. This study provides fundamental data for use in astrochemical models as well as the basis for a future investigation of methyl acetate and methyl propanoate adsorbed in mixed ice environments with water ice.

## Abstract 2

The adsorption of methyl acetate and methyl propanoate has been studied on a graphite surface at 28 K. TPD data show that the desorption energy of both molecules is highly coverage dependent with repulsive interactions being seen at low coverages.

## Graphical abstract

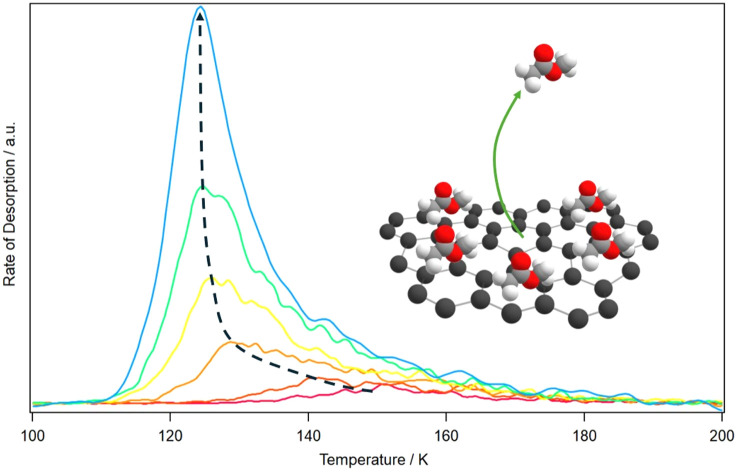

## Introduction

Complex organic molecules (COMs) are defined in astrochemistry as carbon containing species with at least six atoms.^1^ COMs have been identified in the gas phase in environments such as dense molecular clouds, but also in the solid phase as molecular ices frozen out on the surface of dust grains.^2,3^ Surface chemistry has therefore become an integral part of many astrochemical studies, with surface environments such as icy mantles in protoplanetary disks being the focus of observational studies to discover COMs in previously understudied conditions,^2,3^ and many theoretical models including gas–grain exchange processes to explain the observed abundances in interstellar environments.^4,5^

Experimental research therefore plays an important role in astrochemistry, with spectroscopic studies providing valuable reference data to both validate current observations and also give approximate target regions for the search for new species.^3^ Additionally, kinetic data retrieved from temperature programmed desorption (TPD) experiments (both quantitative parameters and qualitative behaviour) can be used in theoretical models to better describe the adsorption and desorption processes that occur in these interstellar environments, for example, in astrochemical simulations of gas–grain chemistry in the interstellar medium or the snowline chemistry of protoplanetary disks.^6–8^

One particularly under-researched phenomenon in this area is the effect of coverage-dependent kinetic desorption parameters. Thin film molecular ices are generally categorised as either sub-monolayer/monolayer or multilayer ices, each obeying certain desorption characteristics according to observed standards in the literature.^9^ This in itself is coverage dependent behaviour, however, previous TPD studies have shown that within the sub-monolayer regime, the kinetic parameters vary considerably due to intermolecular interactions.^10–12^ These effects are often omitted from theoretical models, with many groups opting to treat these sub-monolayer ices with averaged kinetic parameters. This research aims to extend the work of Ligterink *et al.*^13^ in applying their Transition-State-Theory (TST) methods to two test species (methyl acetate and methyl propanoate) to show how the calculated kinetic parameters vary for these highly coverage dependent data sets. This is with a view to using these coverage dependent kinetic parameters in astrochemical simulations of gas–grain exchange. Such models currently account for binding energy distributions derived from different binding sites on the surface, but do not yet explicitly consider the other interactions, such as intermolecular repulsions described here.^14–16^

**Fig. 1:**
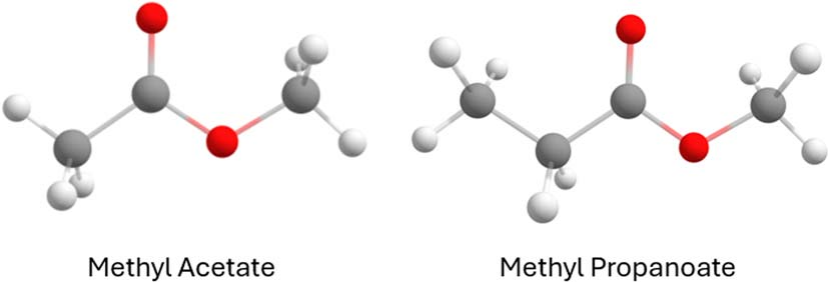
The structure of the simple esters investigated in this study.

Methyl acetate (Fig. 1) is a COM that has been detected in the Orion nebula and on comet 67P/Churyumov–Gerasimenko, making it an appropriate and relevant species for this study.^17^ COMs containing the ester functional group are of particular interest to astrochemists and astrobiologists, as they are believed to act as building blocks for prebiotic species such as amino acids, and could therefore play an early role in the origins of life.^18–20^ Such acetyl amino acids have also been identified in primitive meteorites along with their precursors, including methyl acetate and several other esters.^21,22^ Possible routes of formation and destruction of methyl acetate in the interstellar medium also include propionic acid formation, a likely precursor for the formation of another simple ester–methyl propanoate (Fig. 1).^23^ Although methyl propanoate has not yet been observed in astrophysical environments, with the current state of observational astrochemistry constantly discovering new COMs of similar sizes and with the same functional groups, methyl propanoate has been theorised as a likely candidate for interstellar detection in the future.^24^ As such, experimental studies into these likely candidates are important for validating and supporting both observational and theoretical studies.

In addition to astrochemical studies, the interactions of simple esters and graphitic substrates are also important in atmospheric chemistry. Methyl acetate and methyl propanoate are established pollutants that exist in the Earth's atmosphere as a consequence of industrial processes across the planet.^25,26^ Several studies have shown that these species play a role in atmospheric ozone formation and that their lifetimes in the atmosphere are dictated by oxidation mechanisms with initiators such as chlorine, hydroxyl, and nitro radicals.^25–28^ Despite the conditions of the interstellar medium and the Earth's atmosphere being very different, the same principles of gas phase reactions dictate how these species can interact, and so particulates of polycyclic aromatic hydrocarbons (PAHs) can play the same role as carbonaceous dust grains to provide surfaces for reactive species to accrete onto.^29^ As such, the adsorption and desorption behaviour of COMs on graphitic substrates is also important to atmospheric chemistry, particularly for the simulation of pollutant lifetimes.

Interstellar dust grains are primarily composed of rough and porous carbonaceous or siliceous material and are therefore difficult to simulate reproducibly in a laboratory environment.^30^ Several different surface analogues have been used by experimentalists to date, with highly ordered metallic surfaces including gold,^31,32^ silver,^33,34^ copper,^35,36^ nickel,^37^ and platinum^38^ being generally preferred to give reproducible data that compare well with theoretical models. Highly oriented pyrolytic graphite (HOPG), the surface used in this work, is another commonly used surface,^39–43^ as it provides a uniform carbonaceous surface that gives reproducible results as well as being a semi-metal that obeys the metal-surface selection rule, thus allowing for reflection absorption infrared spectroscopy (RAIRS) to be performed.^44–46^ In addition to adsorption/desorption studies, HOPG has been widely used to investigate the formation of small molecules and COMs, providing the basis for many theoretical studies into both gas and solid phase reaction networks of astrochemical environments.^47–50^ When comparing highly ordered *vs.* rough, porous graphitic surfaces, there is likely to be some variation in the kinetic desorption parameters. However, the methods described here show that these parameters are highly coverage dependent even on the HOPG surface, thus highlighting the need for these kinetic parameters to be given as a range of values as opposed to the discreet values usually reported.

Previous surface science investigations of methyl acetate and methyl propanoate provide reference data for comparison to the work described here. Zahidi *et al.* investigated methyl acetate (as well as methyl formate and ethyl formate) chemisorbed on a Ni(111) surface.^37^ This study used RAIRS and TPD to investigate the annealing of the esters between 140 K and 350 K.^37^ Methyl acetate began thermal decomposition at ∼180 K to produce adsorbed methoxy, acetyl, and carbonyl species. The methoxy species was unstable and immediately dissociated, while the acetyl species was stable up to 300 K. While chemisorbed methyl acetate is not directly comparable to the physisorbed ices investigated here, the thicker, condensed phase, ices studied by Zahidi *et al.*^37^ are relevant to the multilayer ices described here. A study by Sivaraman *et al.* investigated the electron irradiation of methyl acetate ices on a ZnSe surface.^51^ Similar decomposition products to those reported in the work of Zahidi *et al.*^37^ were identified, with the addition of more stable methoxy species. Another infrared investigation focused on methyl acetate condensed on a gold substrate.^52^ It was found that ices grown at 12 K formed an amorphous structure and upon thermal processing to between 110 K and 120 K, the ice underwent an irreversible phase change to a crystalline structure. This hypothesis was supported by the observation of shifts and splitting in the fundamental, overtone, and combination bands of the infrared spectra. Several infrared studies of methyl propanoate adsorbed on non-graphitic substrates (KBr,^24^ ZnSe,^53^ and Cs I^23^) have been undertaken and show spectra that can be compared to the work described here. These studies also reported a phase change for methyl propanoate at 120 K, similar to that observed for methyl acetate.

With the adsorption/desorption processes of COMs from carbonaceous particulates and dust grains being important to the fields of both astrochemistry and atmospheric chemistry, methyl acetate and methyl propanoate ice adsorption and thermal processing on an HOPG surface have been studied using surface science techniques. TPD experiments have been performed to investigate the desorption and crystallisation of the esters on HOPG, and RAIRS experiments have been undertaken to follow how the surface structure of the molecular ices changes when thermally processed. These studies aim to provide reference data to aid in future astrochemical detection efforts and coverage dependent kinetic parameters for use in theoretical models of gas–grain exchange processes.

### Methodology

TPD and RAIRS experiments were performed in a welded stainless-steel ultra-high vacuum (UHV) chamber with a base pressure of ∼2 × 10^−10^ mbar. Ices were grown *via* vapour deposition onto an HOPG surface with carbon–carbon bond lengths of 0.142 nm, interlayer distances of 0.335 nm and angular mosaic spread between layers of <1° (Goodfellows Ltd).^54,55^ The sample was cleaned *via* Scotch tape exfoliation^56,57^ prior to installation in the vacuum chamber and through annealing to 250 K in between experiments. A closed-cycle helium refrigerator (SHI APD) was used to cryogenically cool the HOPG surface to a base temperature of 28 K, monitored using an N-type thermocouple. Ice growth was controlled using high precision leak valves to deposit nanometre thick ices onto the substrate. Exposures are reported in *L*_m_, where 1 *L*_m_ = 1 × 10^−6^ mbar s. Methyl acetate and methyl propanoate were purchased from Sigma Aldrich at ≥99.5% purity and were further purified through several freeze–pump–thaw cycles to remove any dissolved gases.

When growing molecular ices on a cryogenically cooled surface at 28 K, the sticking probability is assumed to be equal to one, meaning that if a gas phase molecule collides with the surface then it will accrete and become part of the ice. This sticking probability is closely related to the surface temperature, and so an increased temperature (up to 120 K in this work) leads to a reduction in the sticking probability, with fewer gas-surface collisions leading to additions to the ice. This was accounted for by increasing the dosing exposures when dosing gases at a higher surface temperature than base temperature. TPD experiments were then used to check that comparable amounts of ice had been deposited at the higher temperature.

TPD experiments were conducted by resistive heating of the HOPG surface at a rate of 0.5 ± 0.01 K s^−1^. Thermal desorption of the esters was monitored using a quadrupole mass spectrometer (QMS, Hiden HAL 301/PIC). The parent ions as well as several fragment masses were followed for each ester, with *m*/*z* = 43 and 57 giving the strongest signals for the desorption of methyl acetate and methyl propanoate respectively.

RAIRS experiments were performed using a Fourier transform infrared (FTIR) spectrometer (Thermo Nicolet 6700) coupled to an external liquid nitrogen cooled mercury–cadmium–telluride (MCT) detector. All spectra were recorded as the coaddition of 256 scans at 4 cm^−1^ resolution, taking 2 minutes 40 seconds to collect each spectrum (approximately 0.63 seconds per scan). Annealing experiments involved heating the HOPG surface in increments of 10 K and holding the temperature for 3 minutes before cooling to base temperature to record the infrared spectrum.

Gaussian16 was used to calculate the rotational constants of the two esters to obtain the principal moments of inertia for use in TST analysis of TPD data (described later). These calculations were run at the CAM-B3LYP/aug-cc-pVDZ level of theory.^58,59^ These calculations treat the esters as monomers in the gas phase, rather than as adsorbates on a surface, but the values are used to predict how the species transition between the two phases, and are therefore a good representation of the desorption process.

## Results and discussion

### RAIRS data

Methyl acetate and methyl propanoate form amorphous ices when vapour deposited on cryogenically cooled surfaces, as shown in the work of Hudson *et al.*^24,60^ and Sivaraman *et al.*^23,52,61^ This work showed that upon thermal processing between 110 K and 120 K, amorphous methyl acetate and methyl propanoate ices underwent an irreversible phase change to a crystalline form. To confirm that the esters grown in this work also show these amorphous and crystalline structures, RAIRS experiments were performed on ices grown at different temperatures, and the assigned bands of the spectra were compared to literature.

**Fig. 2:**
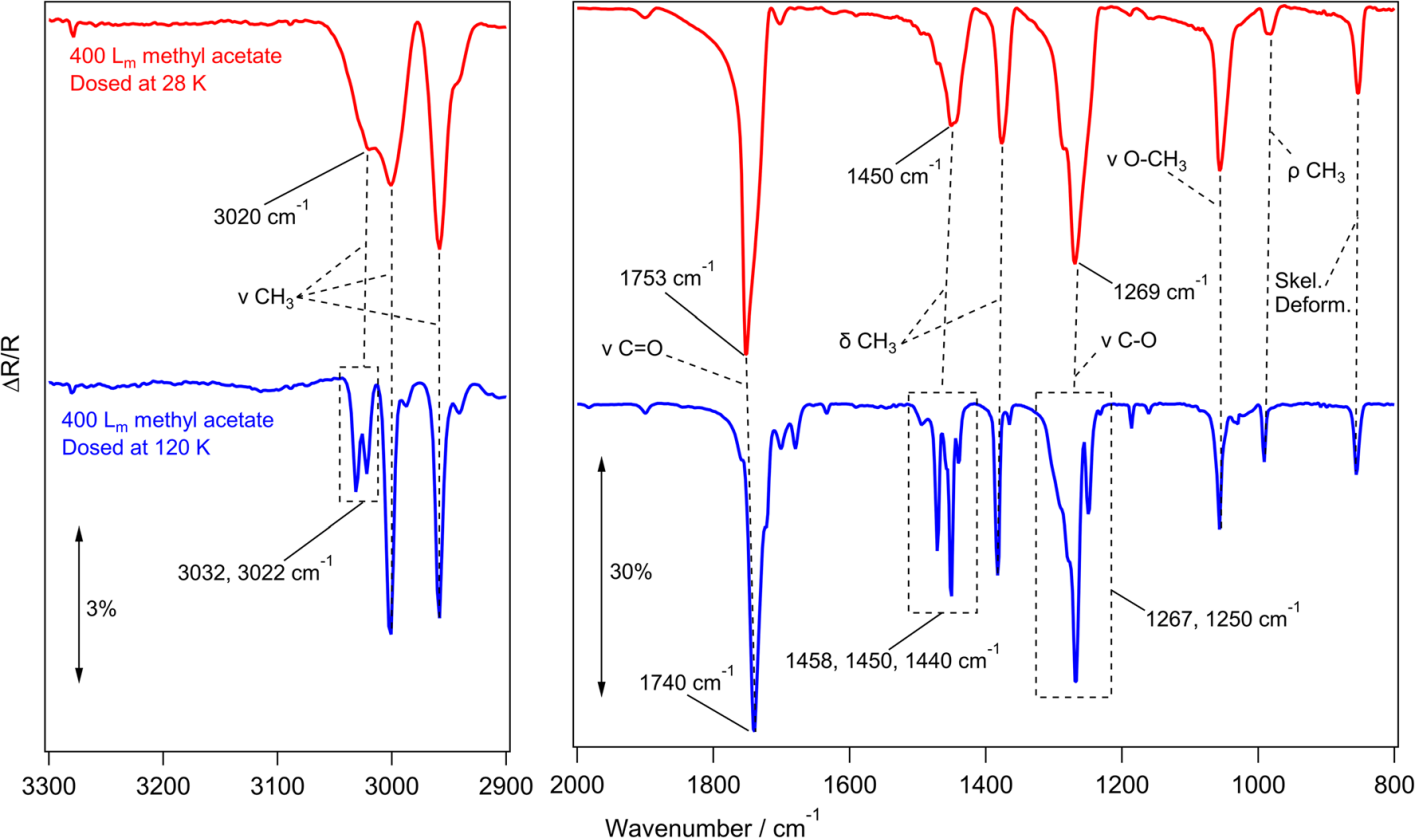
Experimental RAIR spectra of 400 *L*_m_ methyl acetate adsorbed on HOPG at 28 K (red) and 120 K (blue). Selected vibrational modes are highlighted in the spectra. The left hand panel shows the 2900–3300 cm^−1^ wavenumber region and the right hand panel shows the region from 800–2000 cm^−1^. No vibrational bands are seen in other regions of the spectrum.

Fig. 2 shows a series of infrared data recorded for methyl acetate. The red and blue traces show the experimental RAIR spectra of 400 *L*_m_ methyl acetate ices adsorbed on HOPG at 28 K and 120 K respectively.^27^ With the 120 K surface being hotter than the 28 K base temperature, the sticking probability of molecules landing on the surface was decreased, and so the exposures had to be adjusted so that the ice coverages were comparable.

Beginning with the methyl acetate ice grown at 28 K in Fig. 2 (red trace), the most intense peaks at 1753 cm^−1^ and 1269 cm^−1^ are assigned to the carbon–oxygen stretches of the carbonyl and ester functional groups respectively. Other peaks at 1450 cm^−1^ and 1371 cm^−1^ are the asymmetric and symmetric bends of the CH_3_ groups respectively, while the 1057 cm^−1^ peak is assigned to the stretching mode of the O–CH_3_ group. Although much lower in intensity than the other bands, the C–H stretching modes of the CH_3_ groups are also identified at 2958 cm^−1^ and 3001 cm^−1^. The assignments of the observed infrared bands are given in Table 1, along with a comparison to the literature.

**Table:** Assignment of the infrared spectral bands for multilayer methyl acetate adsorbed on HOPG at 28 K, at 120 K and thermally annealed to 120 K. Values from the literature are given for comparison. All values in cm^−1^^a^

Assignment	This work		Literature			
	Dosed at 28 K	Dosed at 120 K	KBr surface^60^		Au Surface^52^	
			Amorphous	Crystalline	Amorphous	Crystalline
*ν*_as_ (C)CH_3_	3020 (sh)	3032	—	—	3022	3031
*ν*_as_ (O)CH_3_		3022	—	—	—	3022
*ν*_as_ (O)CH_3_	3001	3001	2999	3001	2998	3001
*ν*_as_ (C)CH_3_		2987 (sh)	—	—	—	2987
*ν*_s_ (O)CH_3_	2958	2958	2958	2957	2957	2959
*ν*_s_ (C)CH_3_	2941 (sh)	2941 (sh)	—	—	2942	2941
*ν* C=O	1753	1740	1741	1723	1736	1726
*δ*_as_ (O)CH_3_	1495 (sh)	1495	—	—	1491	1495
*δ*_as_ (O)CH_3_	1473 (sh)	1471	—	—	1465	1469
*δ*_as_ (C)CH_3_	1450	1458 (sh)	—	—	—	1457
*δ*_as_ (C)CH_3_		1450	—	—	—	1448
*δ*_s_ (O)CH_3_		1440	1441	1448	1440	1439
*δ*_s_ (C)CH_3_	1377	1383	1372	1381	1369	1365
*ν* C(O)–O	1269	1267, 1250 (sh)	1254	1251	—	1278
*ρ* (O)CH_3_	—	1186	—	—	1192	1186
*ρ* (O)CH_3_	—	1161	—	—	1158	1160
*ν* O–CH_3_	1057	1057	1050	1054	1044	1048
*ρ* (C)CH_3_	—	1032	—	—	—	1036
*ρ* (C)CH_3_	984	991	981	989	977	990
Skel. Def	852	856	851	854	849	853

a Symbols: symmetric stretching (*ν*_s_), asymmetric stretching (*ν*_as_), symmetric bending (*δ*_s_), asymmetric bending (*δ*_as_), and rocking (*ρ*), shoulder (sh).

Moving to the blue trace in Fig. 2, recorded for 400 *L*_m_ methyl acetate grown on HOPG at 120 K, some key differences in the RAIR spectrum compared to the red trace show that this ice has formed as a crystalline ice, as opposed to the amorphous methyl acetate grown at 28 K. The C=O carbonyl peak sharpens and shifts by 13 cm^−1^, from 1753 cm^−1^ to 1740 cm^−1^, and a similar effect is observed for the C–O ester stretch, which shifts by 2 cm^−1^ from 1269 cm^−1^ to 1267 cm^−1^ and splits to reveal a shoulder at 1250 cm^−1^. Similar observations have been previously seen for the crystallisation of methyl formate and ethyl formate,^10,62^ which both showed a sharpening of the C=O and C–O peaks due to a uniform alignment of the dipole moments in the crystalline ices.^10,62^ In addition, several peaks between 3032–2987 (CH_3_ stretching) and 1458–1440 cm^−1^ (CH_3_ bending) take the place of the broad features seen in the amorphous ice.

The infrared spectra recorded for methyl acetate grown at 28 K and 120 K are in good agreement with the transmission IR spectra of methyl acetate on KBr (Yarnall *et al.*)^60^ and with the RAIR spectra on Au (Sivaraman *et al.*),^52^ suggesting that similar amorphous and crystalline ices are being grown in this work.

A 100 *L*_m_ methyl acetate ice was also annealed between 28 K and 130 K in increments of 10 K to follow the thermal processing of the amorphous ice. These spectra are shown in Fig. S1.† No changes were observed in the spectrum between 28 K and 110 K, but upon heating to 120 K, the same shifts and peak splittings that were discussed earlier for Fig. 2 were observed. This suggests that the crystallisation behaviour is the same for thermally processed ices grown at 28 K and those grown at 120 K.

**Fig. 3:**
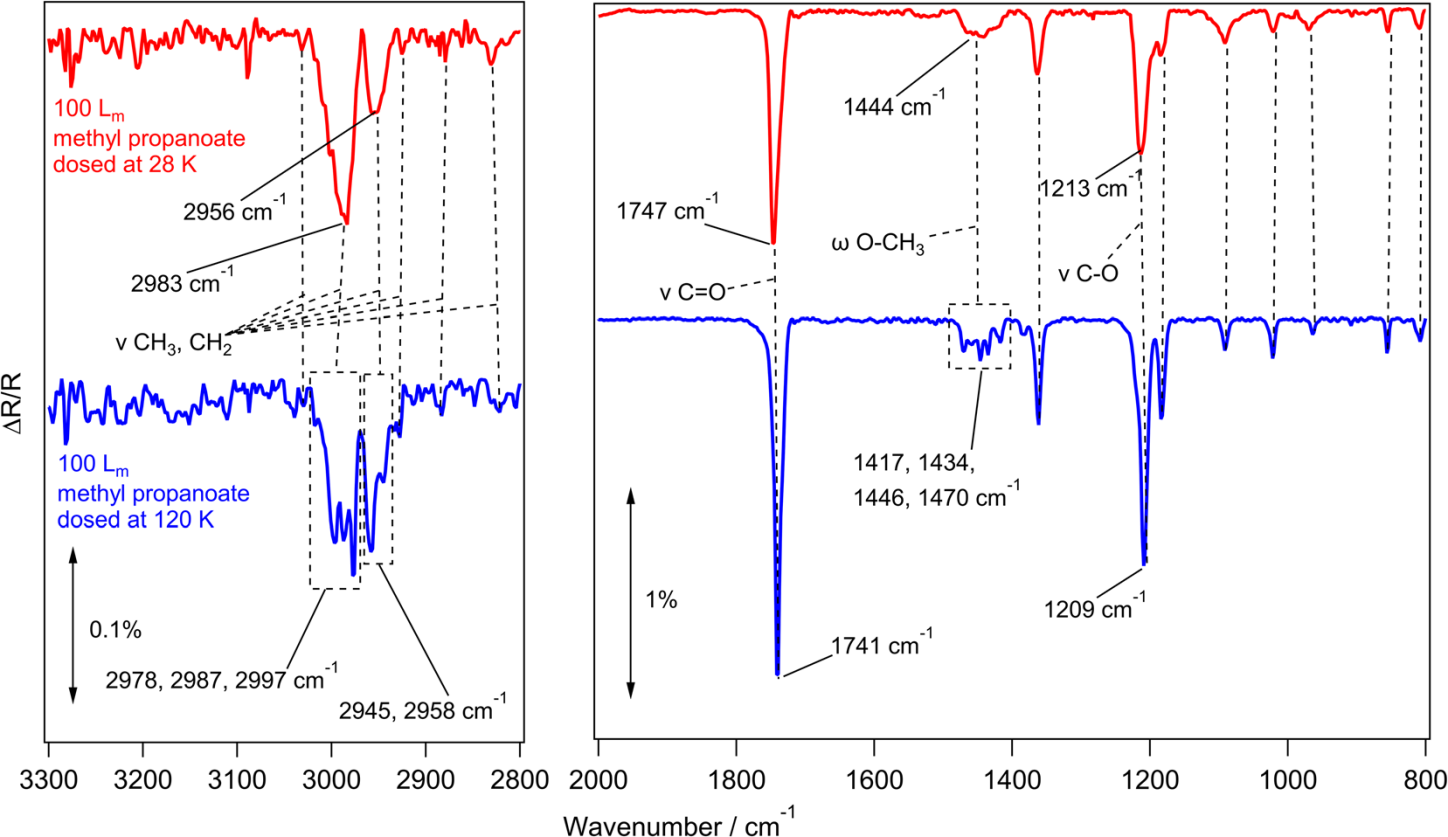
RAIR spectra of 100 *L*_m_ methyl propanoate adsorbed on HOPG at 28 K (red) and 120 K (blue). Selected vibrational modes are highlighted in the spectra. The left hand panel shows the 2800–3300 cm^−1^ wavenumber region and the right hand panel shows the region from 800–2000 cm^−1^. No vibrational bands are seen in other regions of the spectrum.

To investigate the amorphous and crystalline structures of methyl propanoate, the same RAIRS experiments were carried out as previously described. Fig. 3 shows RAIR spectra recorded for the adsorption of 100 *L*_m_ of methyl propanoate on HOPG at 28 K (red trace) and 120 K (blue trace), with the assignments given in Table 2.

**Table:** Assignment of the infrared spectral features for multilayer methyl propanoate adsorbed on HOPG at 28 K, at 120 K and thermally annealed to 120 K. Values from the literature are given for comparison. All values in cm^−1^^a^

Assignment	This work		Literature		
	Dosed at 28 K	Dosed at 120 K	KBr substrate^24^		ZnSe substrate^23^
			Amorphous	Crystalline	
*ν*_as_ (O)CH_3_	3030	3030	—	—	3028
Individual and combination bands of: *ν*_as_ (O)CH_3_, *ν*_as_ (C)CH_3_, *ν*_s_ (O)CH_3_, *ν*_s_ (C)CH_3_, *ν*_as_ CH_2_, and *ν*_s_ CH_2_	3001 (sh), 2983	2997, 2987, 2978	2981	2998, 2995, 2986, 2976	2982
	2956	2958, 2945 (sh)	2953	2958, 2955, 2943	2951
	2930	2928	2925	2929	2927
	2886	2883	2883	2883	2884
	2852	2855, 2848	2851	2855, 2847	2848

*ν* C=O	1747	1741	1741	1735, 1731	1739
δ (C)CH_3_	1444	1471, 1460	1463	1470, 1467, 1460, 1455	1461
*ω* (O)CH_3_ or *ρ* (O)CH_3_		1446, 1435, 1417	1439	1445, 1435, 1421, 1417	1439, 1418

*ω* [CH_3_CH_2_] or *ω* (C)CH_3_	1363	1362	1362	1358	1362
*ρ* [CH_3_CH_2_] or *ν* (O)C–O	1213	1209	1208	1200	1207
*ρ* [OCH_3_]	1184 (sh)	1184	1181	1178	1181
*ω* CH_3_ or *ρ* (C)CH_3_	1092	1092	1090	1091	1090
*ν* O–CH_3_ +	1022	1022	1021	1021	1021
*ν* H_3_C–CH_2_ + *ν* O–CH_3_	970	964	966	963	965
*ν* C–C(O) + *ν* C(O)–O	854	856	854	854	854
*ρ* (C)CH_3_ + *ρ* CH_2_	810	808	808	810, 807	807

a Symbols: symmetric stretching (*ν*_s_), asymmetric stretching (*ν*_as_), bending/scissoring (*δ*), wagging (*ω*), twisting (*τ*), and rocking (*ρ*).

Methyl propanoate behaves similarly to methyl acetate, with the infrared spectra of the amorphous and crystalline forms being distinguished from each other by distinct band shifts and splitting of broad features. This is evident in Fig. 3, with the peak at 1747 cm^−1^ (assigned to the C=O carbonyl stretching mode) sharpening and shifting by 6 cm^−1^ to 1741 cm^−1^. This is also true for the C–O ester stretching mode at 1213 cm^−1^, which shifts to 1209 cm^−1^.

Similarly to methyl acetate, several broad features in the amorphous methyl propanoate spectrum at 2983 cm^−1^, 2956 cm^−1^ (CH_3_ and CH_2_ stretching), and 1444 cm^−1^ (CH_3_ and CH_2_ bending, wagging and/or rocking) split to give individual bands in the crystalline spectrum. While the infrared bands of methyl acetate were straightforward to assign, methyl propanoate proved more difficult, even with reference to the literature studies of Hudson *et al.*^24^ and Sivaraman *et al.*^23^ This is due to the extra CH_2_ group of methyl propanoate, which gives the molecule more flexibility around the skeletal structure, leading to most of the vibrational modes being combination bands of several stretching and bending motions. With this in mind, the assignments provided in Table 2 are only approximate descriptions. Despite this, the band positions of the experimental RAIRS in this work are in good agreement with spectra for amorphous and crystalline methyl propanoate grown on KBr by Hudson *et al.*^24^ and with the amorphous ice grown by Sivaraman *et al.*^23^ Annealing experiments were also performed for a 100 *L*_m_ methyl propanoate ice grown at 28 K, with similar results showing the amorphous to crystalline phase change at 120 K (Fig. S2†).

### TPD data

**Fig. 4:**
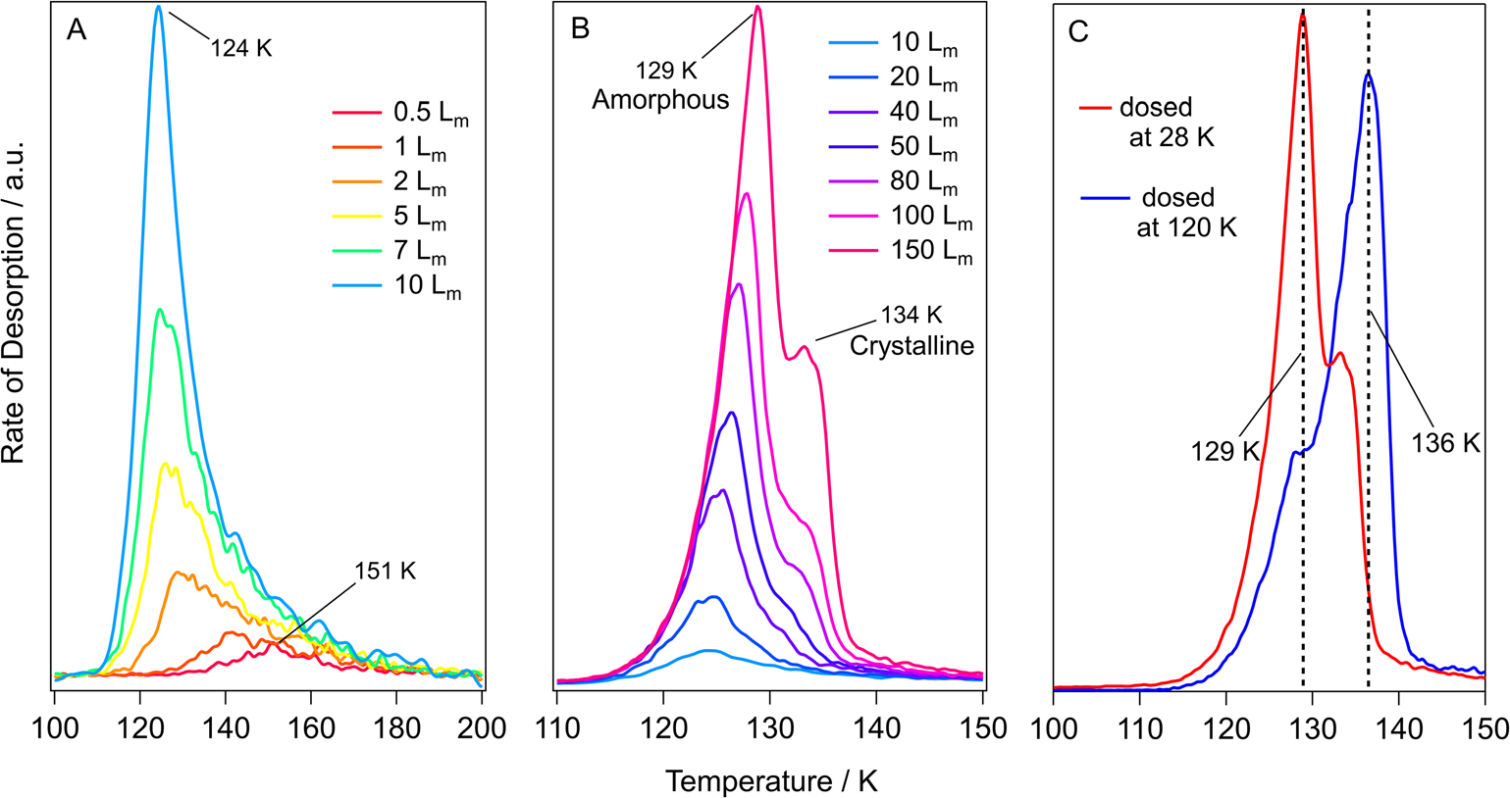
TPD spectra for varying exposures of methyl acetate adsorbed on HOPG. (A) Exposures from 0.5 *L*_m_–10 *L*_m_ dosed at 28 K. (B) Exposures from 10 *L*_m_ – 150 *L*_m_ dosed at 28 K. (C) A comparison of 150 *L*_m_ methyl acetate ices grown at 28 K and 120 K.

TPD data for pure methyl acetate ices grown on HOPG at 28 K are shown in Fig. 4. The features in Fig. 4 show low temperature desorption, between 124 K and 151 K, suggesting that methyl acetate is physisorbed on the graphite surface. This is in agreement with the RAIR spectra shown earlier. Fig. 4A shows TPD spectra for low exposures of 0.5 *L*_m_ to 10 *L*_m_. These spectra show a single, broad, desorption feature with a peak temperature that decreases as the exposure increases, from 151 K at 0.5 *L*_m_ to 124 K at 5 *L*_m_. This decreasing peak temperature with increasing exposure has been previously observed for studies of methyl formate and ethyl formate adsorbed on HOPG and has been assigned to intermolecular repulsions between the sub-monolayer adsorbates.^10,62^ These adsorbate–adsorbate repulsions were first reported for hydrogen and carbon monoxide adsorbed on tungsten surfaces by Adams^63^ and then later described for benzene in three separate studies on palladium,^64^ silver,^65^ and silicate^66^ surfaces. The increased coverage in the sub-monolayer ices leads to more adsorbate–adsorbate repulsions which manifests in the TPD spectra as a decreasing peak temperature due to a decreased binding energy. Exposures of 5 *L*_m_ to 20 *L*_m_ (Fig. 4A and B) show a constant peak temperature of 124 K, suggesting that these exposures give rise to monolayer adsorption and show first-order desorption kinetics.^9^ Higher exposures of 40 *L*_m_ to 150 *L*_m_ (Fig. 4B) display a shared leading edge and increasing peak temperature from 125 K to 129 K, suggesting multilayer desorption following zero-order kinetics.^9^

Fig. 4B shows that exposures of 50 *L*_m_ and higher lead to the formation of a high temperature shoulder at around 134 K, that increases in intensity as the coverage increases. This shoulder most likely arises due to the desorption of crystalline methyl acetate, the formation of which was seen previously in the RAIRS data. This was further investigated by growing a 150 *L*_m_ methyl acetate ice at 120 K (accounting for the change in sticking probability) to compare the differences in the desorption profiles. This is shown in Fig. 4C, with the same desorption features being present for both ices, but with the high temperature shoulder having much larger intensity. This confirms the assignment of the high temperature shoulder, seen in Fig. 4B, to the desorption of crystalline methyl acetate. RAIRS data (shown earlier) clearly showed the formation of crystalline methyl acetate following dosing at 120 K.

**Fig. 5:**
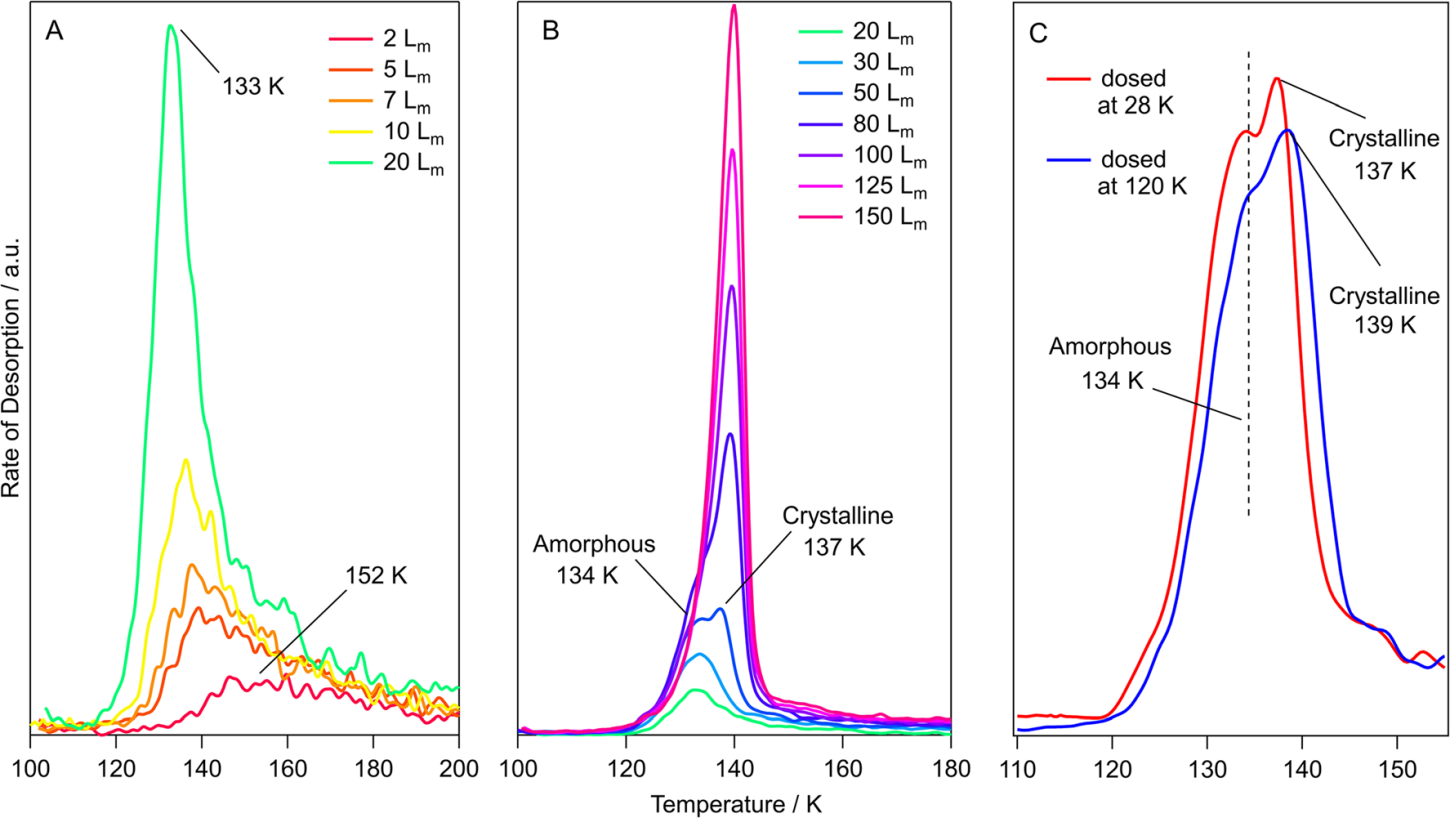
TPD data for varying exposures of methyl propanoate adsorbed on HOPG. (A) Exposures from 2 *L*_m_–20 *L*_m_ dosed at 28 K. (B) Exposures from 20 *L*_m_–150 *L*_m_ dosed at 28 K. (C) A comparison of 50 *L*_m_ methyl propanoate ices grown at 28 K and 120 K.

The structure of methyl propanoate differs from methyl acetate only by an extra CH_2_ group on the ester chain, as shown in Fig. 1. As such, the desorption and phase change behaviour of the two esters are expected to be similar. TPD data for pure methyl propanoate adsorbed on HOPG are shown in Fig. 5. As for methyl acetate, the low desorption temperatures suggest physisorption of the molecule on the HOPG surface. Fig. 5A and B show the desorption of increasing exposures of methyl propanoate, with very similar trends to those seen for methyl acetate (decreasing peak temperatures from repulsive interactions and a shared leading edge at higher coverages). However, an important distinction between the two esters is the slightly higher temperatures at which methyl propanoate desorbs from the HOPG surface, desorbing between 2 and 10 K higher than corresponding exposures of methyl acetate. This higher desorption temperature arises because methyl propanoate is the larger of the two species.

The desorption of the 50 *L*_m_ ice (Fig. 5B) shows two distinct desorption features at 134 K and 137 K, which are assigned to the desorption of amorphous and crystalline methyl propanoate, in line with the conclusions from the RAIRS data and with the observations seen for methyl acetate. As for methyl acetate, methyl propanoate ices were also grown at a higher temperature (120 K) to investigate the formation and desorption of crystalline methyl propanoate (Fig. 5C). Unlike the spectra recorded for methyl acetate, both the base temperature (red trace) and high temperature (blue trace) experiments in Fig. 5C show very similar desorption profiles. This is a consequence of the higher desorption temperature of multilayer methyl propanoate, where the desorption of amorphous methyl propanoate occurs at a very similar temperature to the phase change. Hence, the majority of the methyl propanoate ice crystallises before desorption and therefore only the desorption of crystalline ice is seen in the TPD spectra.

### Kinetic analysis of TPD data

In order to determine the kinetic parameters for the desorption of methyl acetate and methyl propanoate from the HOPG surface, Polanyi–Wigner (PW) and TST analysis were undertaken for the data shown in Fig. 4 and 5. These methods have been described in detail previously by Burke *et al.*^9^ and Ligterink *et al.*^13^ respectively, but will also be described here briefly.

The analysis begins with identifying the point at which the ice growth changes from monolayer growth to multilayer growth, as the kinetic parameters of these two regimes are calculated using different methods (PW and TST). These two different analysis methods are required due to the coverage dependency of the energy of desorption and pre-exponential factor for the lowest exposure ices, described in detail later. The PW analysis treats these two parameters as constant, and is therefore only appropriate for describing the higher exposure, non coverage dependent, ices. However, TST can be used to calculate the energy of desorption and pre-exponential factors for each individual sub-monolayer exposure, showing exactly how these parameters change as a function of coverage.

**Fig. 6:**
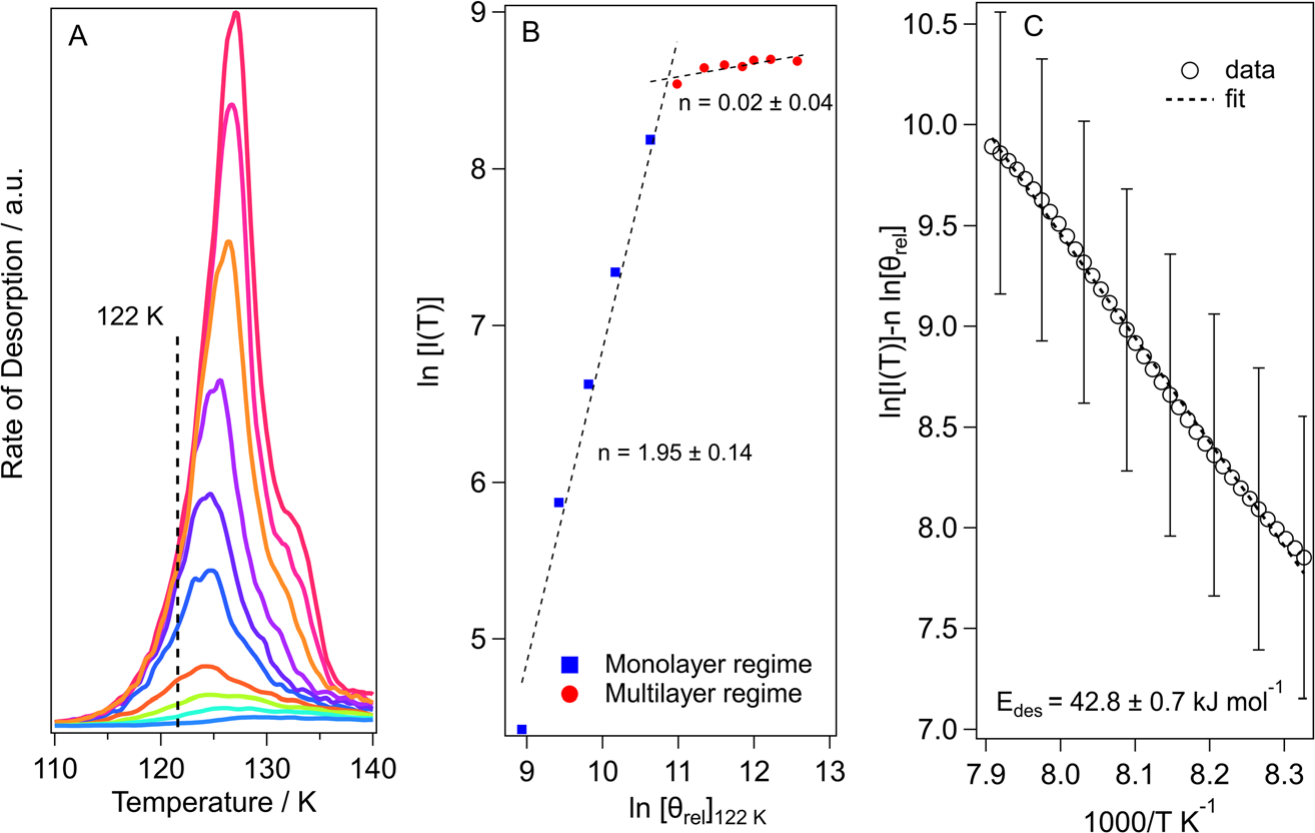
(A) TPD traces of increasing exposures (2 L_*m*_ to 100 L_*m*_) of methyl acetate ice desorption from an HOPG surface. (B) Order of desorption plot constructed from the QMS signal intensity at 122 K from the data in panel (A). (C) Multilayer desorption energy plot for the 50 *L*_m_ data in panel (A).

The threshold for the beginning of multilayer ice growth is identified by comparing the desorption profiles of different ice thicknesses (Fig. 6A for methyl acetate and Fig. S3A† for methyl propanoate) so that plots can be constructed to determine the order of desorption (Fig. 6B and S3B†) from the relative coverages at a common desorption temperature for each TPD trace. This allows the exposure at which the change from monolayer to multilayer ice growth occurs to be determined very easily.

The starting point of this analysis is the Polanyi–Wigner equation (eqn (1)):^67,68^1
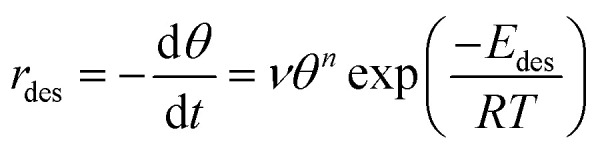
where *r*_des_ is the rate of desorption, *θ* is the adsorbate coverage, *ν* is the pre-exponential factor for desorption, *n* is the order of desorption, *E*_des_ is the desorption activation energy, *R* is the gas constant, *T* is the substrate temperature and *t* is time. Since the rate of change of coverage with time is linked, *via* a linear heating rate, to the rate of change of coverage with temperature (which is proportional to the signal intensity in the mass spectrometer, *I*(*T*)), then eqn (1) becomes:2
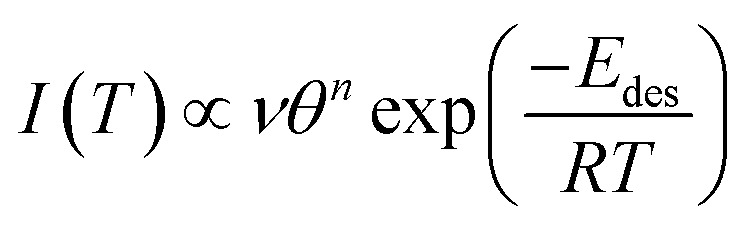


Rearranging this equation and taking logarithms, and noting that only relative coverage *θ*_rel_ can be measured in the experiments described here, eqn (2) becomes:3
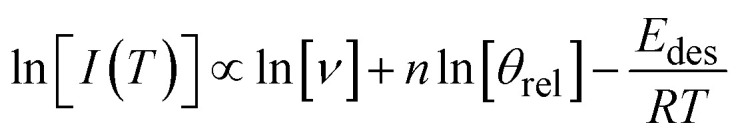


The desorption order can then be obtained by plotting a graph of ln[*I*(*T*)] *versus* ln[*θ*_rel_] for a series of TPD curves of varying initial exposure at a fixed temperature. The gradient of this plot gives the order of desorption, *n*. These plots are shown in Fig. 6B for methyl acetate and Fig. S3B† for methyl propanoate.

The two regimes seen in Fig. 6B clearly indicate monolayer and multilayer desorption, with the change from monolayer to multilayer desorption for methyl acetate occurring at ∼30 *L*_m_ (Fig. 6B) and that for methyl propanoate (Fig. S3B†) occurring at ∼25 *L*_m_. The monolayer order of desorption for methyl acetate from Fig. 6B is equal to 1.95. A desorption order of 2 would suggest second order desorption which can only be achieved *via* recombinative desorption. Since we know from the RAIRS data that the methyl acetate is physisorbed molecularly on the surface, then we know that dissociative adsorption does not occur. Hence, this high order of desorption occurs due to repulsive intermolecular interactions that occur between the sub-monolayer adsorbates, as already discussed. For methyl propanoate, the plot in Fig. S3B† gives a monolayer order of desorption of 1.45. For the same reasons as for methyl acetate, this again is not a realistic value. In light of this, the order of desorption for the sub-monolayer coverages of both esters was set to *n* = 1, the ideal value for first order desorption, in agreement with analysis undertaken for other molecules.^11,13,62^ The orders of desorption for multilayer methyl acetate and methyl propanoate are close to zero (*n* = 0.02 and 0.14 respectively), as expected for multilayer desorption.

The calculated multilayer order of desorption was then used to determine the energies of desorption (Fig. 6C) and pre-exponential factors for multilayer methyl acetate (30 *L*_m_ to 150 *L*_m_) and methyl propanoate (30 *L*_m_ to 150 *L*_m_). Rearrangement of eqn (3) gives eqn (4):4
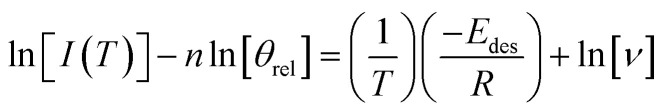


Hence a plot of ln[*I*(*T*)] − *n* ln [*θ*_rel_] *versus* 1/*T* gives the energy of desorption from the gradient of the graph. An example desorption energy plot for multilayer methyl acetate is shown in Fig. 6C. The multilayer desorption energy is determined to be 42.8 ± 0.7 kJ mol^−1^ for an exposure of 50 *L*_m_. Determining the desorption energy for all multilayer exposures of methyl acetate allows an average multilayer desorption energy for both amorphous and crystalline methyl acetate to be calculated, as shown in Table 3. Crystalline multilayer values are determined by peak fitting the TPD data to separate the two phases. An example of the peak fitting for methyl acetate is shown in Fig. S4.† For methyl propanoate, it is only possible to determine a desorption energy for the crystalline multilayer, also shown in Table 3, since the ice becomes crystalline prior to desorption as already discussed. Once the order and energy of desorption have been calculated, these can then be put into the Polanyi–Wigner equation to calculate the pre-exponential factor for multilayer desorption. The calculated pre-exponential values are also given in Table 3.

Kinetic parameters for methyl acetate and methyl propanoate adsorbed on HOPG at 28 K. Monolayer values are given as a range. Literature values are given for comparison

**Table:** 

Methyl acetate	Desorption order	*E*_des_/kJ mol^−1^	Pre-exponential factor, *ν*
Monolayer (amorphous)	1^a^	57.1 ± 0.4 → 47.2 ± 0.3	3.1 × 10^19±0.2^ → 1.6 × 10^19±0.1^ s^−1^
Multilayer (amorphous)	0.02 ± 0.04	43.5 ± 0.9	4.2 × 10^32±0.4^ molec cm^−2^ s^−1^
Multilayer (crystalline)	0^b^	45.8 ± 0.9	1.6 × 10^32±0.4^ molec cm^−2^ s^−1^
Ligterink *et al*^13^	—	64.9^c^	4.1 × 10^19^ s^−1^

a The desorption order for monolayer ices was set equal to 1, as described in the text.

b The order of desorption of crystalline multilayer was assumed to be 0.

c Literature values for *E*_des_ and *ν* were reported by Ligterink *et al*.^13^ using TST analysis on the TPD data from Zahidi *et al*.^37^

**Table:** 

Methyl propanoate	Desorption order	*E*_des_/kJ mol^−1^	Pre-exponential factor, *ν*
Monolayer (amorphous)	1^a^	57.0 ± 0.1 → 51.0 ± 0.1	7.7 × 10^19±0.1^ → 4.4 × 10^19±0.1^ s^−1^
Multilayer (crystalline)	0.14 ± 0.16	45.7 ± 0.9	8.7× 10^29±0.4^ molec cm^−2^ s^−1^
Hudson *et al.*^24^ (crystalline)	—	51 ± 5.1	—

The PW analysis works well for multilayer ices but does not work for monolayer ices if the desorption energy and pre-exponential factor are coverage dependent. As already described, for both monolayer methyl acetate and methyl propanoate the desorption temperature decreases with increasing exposure, due to the presence of repulsive interactions. In this case, both the desorption energy and pre-exponential factor change with exposure, which means that eqn (4) cannot be used to determine the desorption energy accurately. Hence an alternative method of analysis must be used to determine the monolayer desorption energies and pre-exponential factors. As such, these parameters were determined using the TST method, as described in detail elsewhere.^13,47^

The TST method can be applied to the experimental data to first determine *ν* using the values of the translational and rotational degrees of freedom of the adsorbed species:^13,47^5
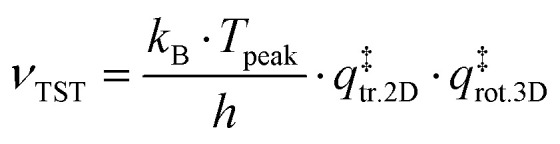
where *k*_B_ is the Boltzmann constant, *T*_peak_ is the peak desorption temperature from the experimental TPD data, *h* is Planck's constant, 
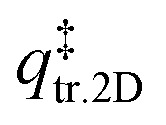
 is the 2D translational partition function and 
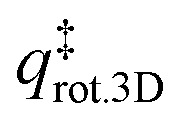
 is the 3D rotational partition function. The 2D translational partition function is calculated as follows:6
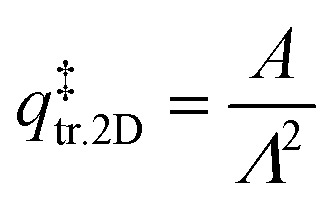
7
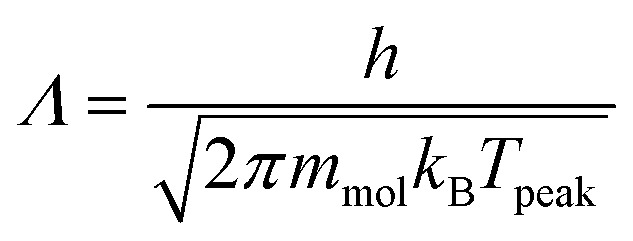
where *A* is the surface area of each adsorbed molecule (fixed at 10^−19^ m^2^, from the assumed number of binding sites for small molecules),^13^*Λ* is the thermal wavelength of the species and *m*_mol_ is the mass of one adsorbate.

The 3D translational partition function is calculated as follows:8
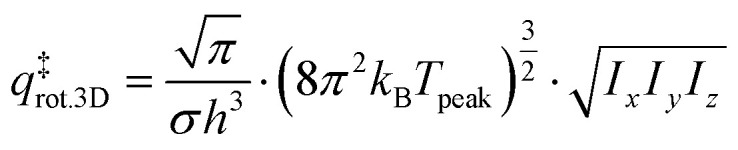
where *σ* is the symmetry factor of the species obtained from first principles of molecular symmetry (Table S1†) and *I*_*x*,*y*,*z*_ are the principal moments of inertia for rotation of the adsorbate, calculated computationally at the CAM-B3LYP/aug-cc-pVDZ level of theory (Table S1†). The calculated pre-exponential factor (*ν*_TST_) can then be used in the Polanyi–Wigner equation (eqn (1)) to calculate *E*_des_.

**Fig. 7:**
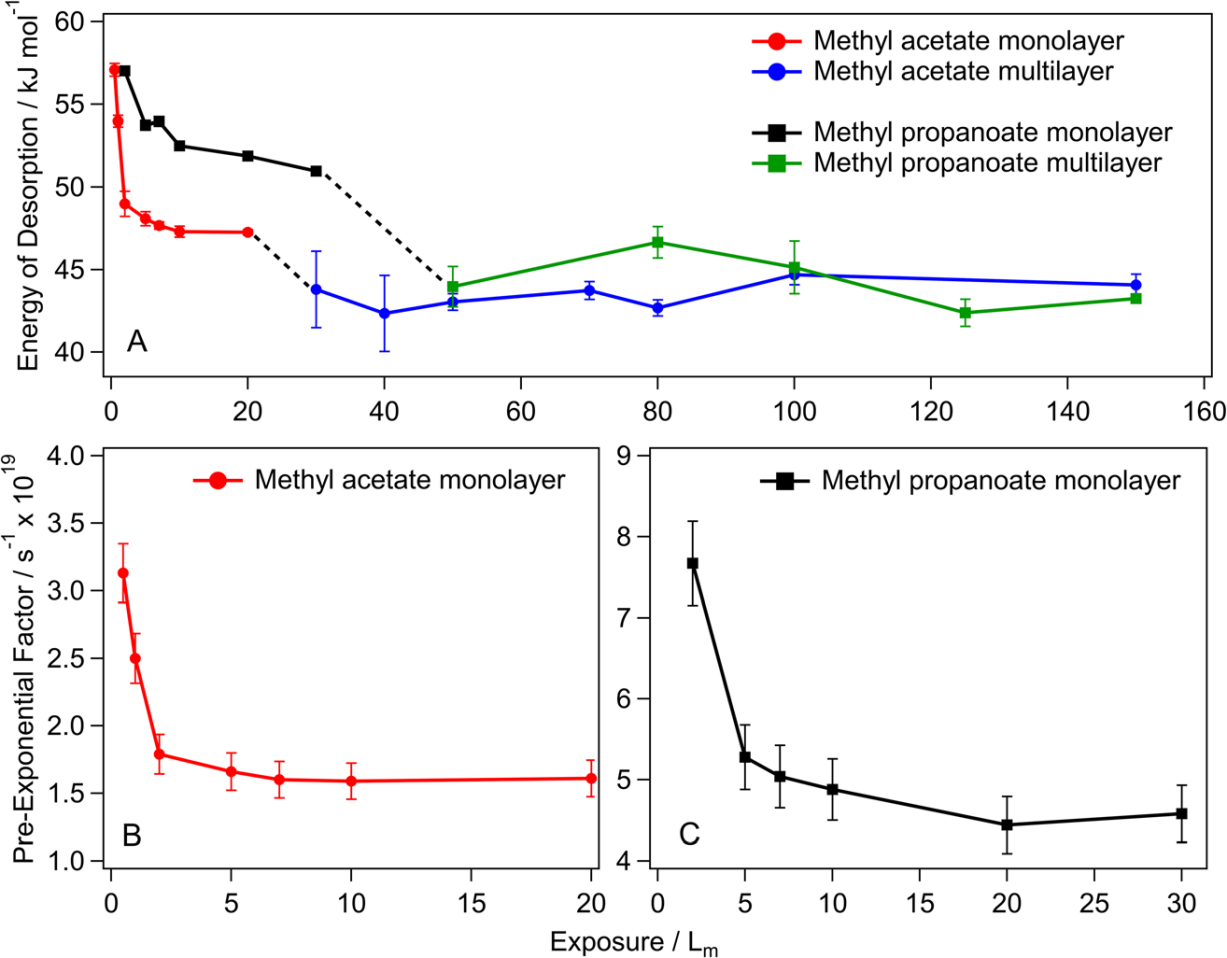
Kinetic parameters for methyl acetate and methyl propanoate adsorbed on HOPG at 28 K. (A) The energy of desorption as a function of exposure. (B) The pre-exponential factor for the methyl acetate monolayer exposures. (C) The pre-exponential factor for the methyl propanoate monolayer exposures.

For methyl acetate and methyl propanoate, the kinetic parameters are highly exposure dependent, with the sub-monolayer desorption energies and pre-exponential factors gradually decreasing as the exposure increases. Fig. 7 shows this exposure dependence for both methyl acetate and methyl propanoate, with the methyl acetate sub-monolayer energies (panel A) decreasing from 57.1 ± 0.4 kJ mol^−1^ (0.5 *L*_m_) to 47.2 ± 0.3 kJ mol^−1^ (20 *L*_m_) – a decrease of 10 kJ mol^−1^. For 30 *L*_m_ and above, the multilayer forms and the energy of desorption plateaus to reach the coverage independent value of 43.5 ± 0.9 kJ mol^−1^. The same trend is shown for the pre-exponential factors (Table 3 and Fig. 7B), with a decrease in the value of the pre-exponential factor for methyl acetate as the exposure increases from 0.5 *L*_m_ to 20 *L*_m_ and a constant value for multilayer ices (not shown).

Similar trends in the desorption energies and pre-exponential factors are observed for methyl propanoate, with energy of desorption (Fig. 7A) and pre-exponential (Fig. 7C) values decreasing from 57 ± 0.1 kJ mol^−1^ and 7.7 × 10 ^19^ s^−1^ (2 *L*_m_) to 51 ± 0.1 kJ mol^−1^ and 4.4 × 10 ^19^ s^−1^ (30 *L*_m_) respectively.

The strong coverage dependence of the kinetic parameters for the lowest coverages is due to the repulsive interactions that govern the changing peak temperatures observed in Fig. 4A and 5A. These have been observed previously for COMs such as acetaldehyde, acetonitrile and benzene,^11,12,48^ as well as for other simple esters such as methyl formate and ethyl formate.^10,62^ From an astrochemical perspective, this coverage dependence is an important factor to consider when modelling the desorption of COMs from icy mantles, as a single set of kinetic parameters may not be sufficient to fully simulate the gas–grain mechanics, particularly when accounting for astronomical timescales of thousands of years.^14–16^

Although there have been several studies on the desorption behaviour of methyl acetate, only one monolayer energy of desorption has been reported and no values are available for the multilayer parameters. Ligterink *et al.* used TST and Redhead analysis to determine desorption energies for TPD data from previous studies across the literature,^13^ some of these values are presented in Table 4 for comparison with the coverage dependent parameters calculated in this work.^13^ For methyl acetate, they used TPD data from Zahidi *et al.* (values shown in Table 3).^13,37^ Their reported pre-exponential factor is similar to that calculated in this work, but their energy of desorption is considerably higher (an increase of 7.9 kJ mol^−1^). However, this is not unsurprising as the methyl acetate in that study is chemisorbed on Ni(111), whereas it is physisorbed on the HOPG surface used here.

**Table:** Monolayer kinetic desorption parameters for a selection of COMs reported as coverage dependant values or analysed using TST

Molecule	Mr	*E*_des_/kJ mol^−1^	Pre-exponential factor, *ν*/s^−1^	Ref.
Acetonitrile	43	48.3 → 44.7	3.9 × 10^17^ → 3.7 × 10^17^	69
		50.0 → 35.0	1 × 10^13^	12
Acetaldehyde	44	49.3	1.6 × 10^18^	13
		36.7	7.7 × 10^17^	13
		35.9	7.2 × 10^17^	13
Methyl formate	60	38.0	2.2 × 10^18^	13
Methyl acetate	74	57.1 ± 0.4 → 47.2 ± 0.3	3.1 × 10^19±0.2^ → 1.6 × 10^19±0.1^	This work
Ethyl formate	74	45.7	1.3 × 10^19^	13
Methyl propanoate	88	57.0 ± 0.1 → 51.0 ± 0.1	7.7 × 10^19±0.1^ → 4.4 × 10^19±0.1^	This work

The energy of desorption of crystalline methyl propanoate calculated in this work is 5.3 kJ mol^−1^ lower than that reported by Hudson *et al.*^24^ This difference is only just outside of the error bounds provided in that study. In that work, the energy of desorption was calculated *via* the construction of a Clapeyron plot from the vapour pressure of methyl propanoate. As such, the pre-exponential factor was not determined and can therefore not be compared to this work.

As there are no literature values for the kinetic desorption parameters of monolayer physisorbed methyl acetate or methyl propanoate, the values determined here have been compared to those of similar sized and functionalised molecules as well as the re-analysed data from Ligterink *et al.*^13^ These are presented in Table 4.

Starting by comparing the four esters (methyl formate, methyl acetate, ethyl formate, and methyl propanoate), there is a clear correlation between the kinetic parameters and molecular size, with the smallest (methyl formate) having the lowest desorption energy and pre-exponential factor. While methyl acetate and methyl propanoate have very similar energies of desorption for their respective lowest coverage, the pre-exponential factor for methyl propanoate is greater by over a factor of two, highlighting the importance of calculating and including both kinetic parameters in these studies, as opposed to assuming a standard value for the pre-exponential factor – a common practice in laboratory surface science studies. Acetonitrile and acetaldehyde values have also been included in Table 4 to show how smaller species compare. As expected, these generally have lower energies of desorption and pre-exponential factors.

## Conclusions

TPD and RAIRS experiments have been carried out for methyl acetate and methyl propanoate adsorbed on HOPG at 28 K. Both species display strongly coverage dependent behaviour, with peak desorption temperatures decreasing as the exposure increases up to the point at which monolayer ice growth gives way to multilayer ice growth. Due to this coverage dependence, it was not possible to use leading edge analysis to determine monolayer desorption for either molecule and an alternative method, TST, was instead used to determine coverage dependent values of the desorption energy and pre-exponential factor. For methyl acetate, the energy of desorption decreases from 57.1 ± 0.4 kJ mol^−1^ at the lowest exposure (0.5 *L*_m_), down to 47.2 ± 0.3 kJ mol^−1^ at 20 *L*_m_, before converging to the multilayer value of 43.5 ± 0.9 kJ mol^−1^. The pre-exponential factors follow the same coverage dependent trend in the monolayer regime, starting at 3.1 × 10^19±0.2^ s^−1^ for the lowest exposure (0.5 *L*_m_) and then decreasing to 1.6 × 10^19±0.1^ s^−1^ for 20 *L*_m_. The same trends are observed for methyl propanoate, with the energy of desorption and pre-exponential factors starting high for the lowest exposure (57 ± 0.1 kJ mol^−1^ and 7.7 × 10^19^ s^−1^ at 2 *L*_m_) and then decreasing to 51.0 ± 0.1 kJ mol^−1^ and 4.4 × 10^19^ s^−1^ at 30 *L*_m_. Neither the TPD nor RAIRS data suggest any thermal chemistry of the molecular ices upon annealing, in line with what is expected for physisorbed species. Both species undergo a phase transition upon thermal processing which can be observed in both the RAIRS (through sharpening of the carbonyl stretching mode and splitting of the CH_2_/CH_3_ bands) and TPD experiments (as the formation of shoulder peaks). With the behaviour of these simple esters characterised as pure ices adsorbed on HOPG, future studies can incorporate water surfaces to investigate how these species interact in more astronomically relevant environments containing water ices and this will be the subject of a future publication.

Kinetic desorption parameters, such as those calculated in this work, have been previously used in astrochemical models to predict the desorption behaviour of ices under non-linear heating rates on timescales more relevant to the interstellar medium.^70^ These models demonstrate the importance of accurate kinetic parameters, with species such as methyl formate, glycolaldehyde and acetic acid desorbing on vastly different timescales under the same heating conditions despite being isomers of each other.^70^ With this in mind, the coverage dependence of the kinetic parameters for these esters (and similar species) can now be incorporated into these models to improve how the lowest coverages of COMs are treated in these simulations.

## Data availability

Raw experimental data and computational output files are available and can be found at the University of Sussex data repository; at http://dx.doi.org/10.25377/sussex.26058322.

## Author contributions

Jack E. Fulker: experiments, theoretical calculations, writing – original draft, data interpretation and commented on the paper. Wendy A. Brown: initiation and management of the project, writing – original draft, data interpretation and commented on the paper.

## Conflicts of interest

The authors declare that they have no known competing financial interests or personal relationships that could have appeared to influence the work reported in this paper.

## Supplementary Material

RA-014-D4RA04466E-s001
