# Supplementary material for: Surface science studies of the coverage dependent adsorption of methyl acetate and methyl propanoate on graphite
Source: RSC Adv. 2024 Nov 5;14(48):35373–85. doi: 10.1039/d4ra04466e (PMC11537193; doi:10.1039/d4ra04466e)
Supplement: RA-014-D4RA04466E-s001 [file RA-014-D4RA04466E-s001.pdf]

Supplementary Information (SI) for RSC Advances.
This journal is © The Royal Society of Chemistry 2024

Surface science studies of the coverage dependent adsorption of methyl acetate and methyl propanoate on graphite

Jack E. Fulker and Wendy A. Brown

Department of Chemistry, University of Sussex, Falmer, Brighton, BN1 9QJ, UK

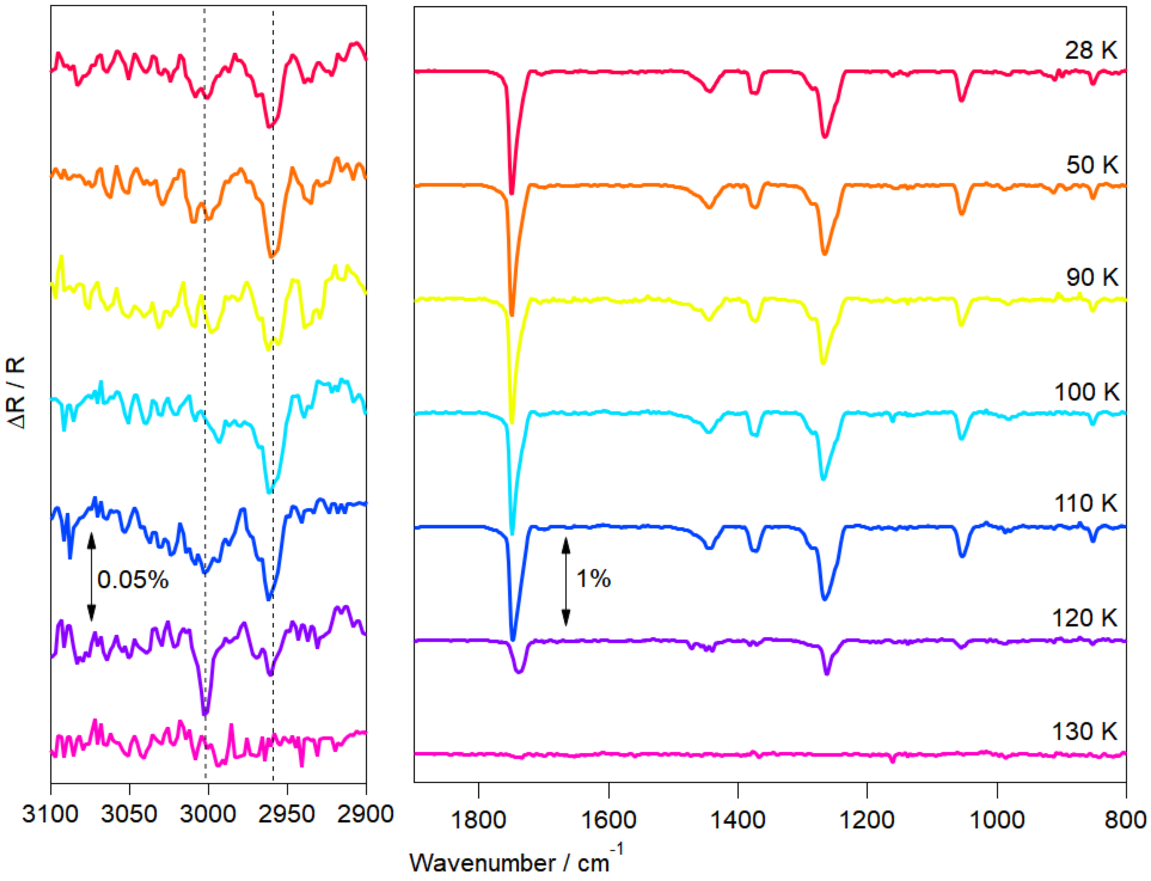

Figure S1: *RAIR spectra for the annealing of a 100 L_m methyl acetate ice adsorbed on HOPG . The ice was grown at 28 K and heated at a rate of 0.5 K s^{-1} in increments of 10 K, holding at each temperature for 3 minutes before cooling back to base temperature to record a spectrum. The left hand panel shows the 2900 – 3100 cm^{-1} wavenumber region and the right hand panel shows the region from 800 – 1900 cm^{-1}. No vibrational bands are seen in other regions of the spectrum.*

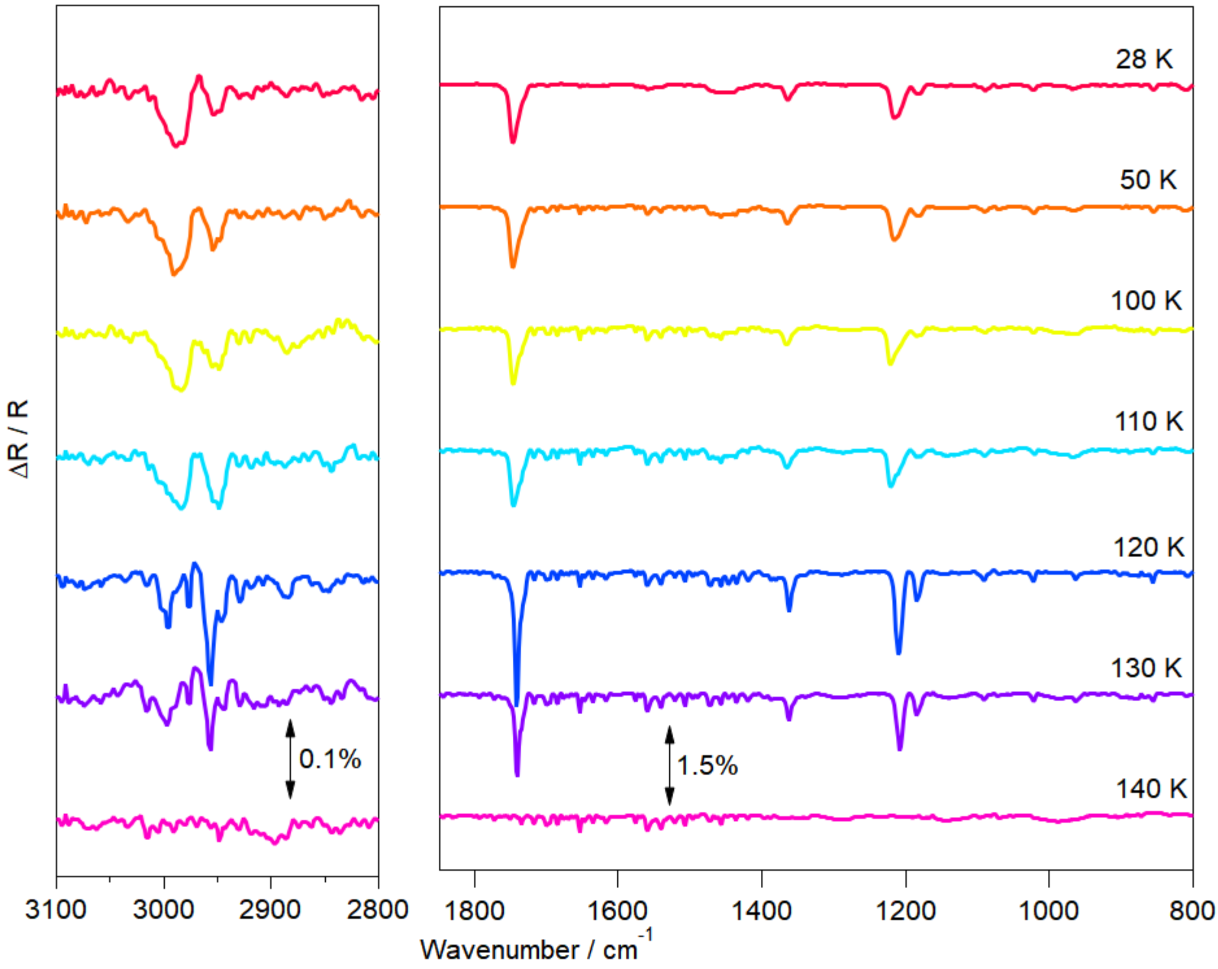

Figure S2: *RAIR spectra for the annealing of a 100 L_m methyl propanoate ice adsorbed on HOPG. The ice was grown at 28 K and heated at a rate of 0.5 K s^{-1} in increments of 10 K, holding at each temperature for 3 minutes before cooling back to base temperature to record a spectrum. The left hand panel shows the 2800 – 3100 cm^{-1} wavenumber region and the right hand panel shows the region from 800 – 1900 cm^{-1}. No vibrational bands are seen in other regions of the spectrum.*

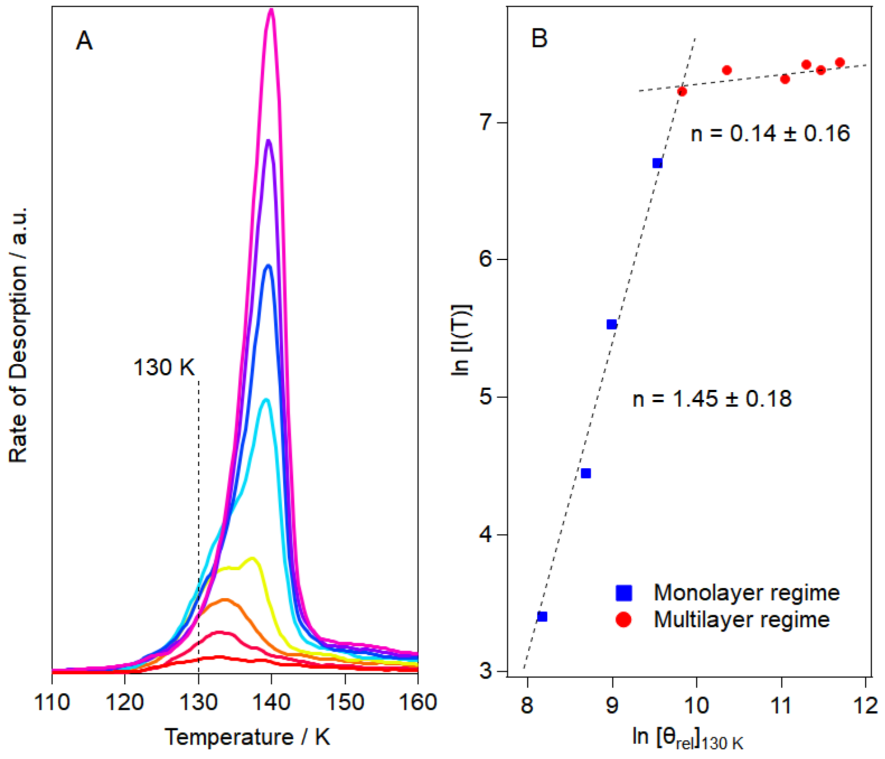

Figure S3: *(A) TPD data for methyl propanoate adsorbed on HOPG with the common temperature of 130 K labelled. (B) An example order plot for the desorption at 130 K.*

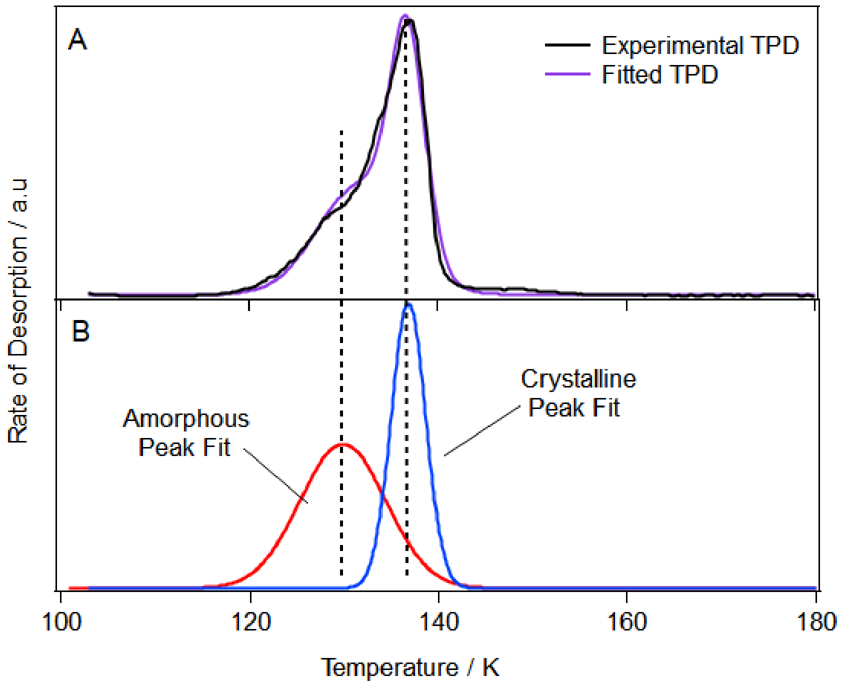

Figure S4: *An example peak fit for the separation of amorphous and crystalline methyl acetate desorption for a 180 L_m ice grown at 120 K on HOPG. (A) The experimental data compared to the fitted trace. (B) The separated amorphous and crystalline peaks.*

Table S1: *Molecular constants used in the transition-state-theory analysis.*

	Mass / amu	Principle moments of inertia / amu Å^2			Symmetry factor (σ)
		I_x	I_y	I_z	
Methyl acetate	74	163.83	121.04	49.16	1
Methyl propanoate	88	278.46	235.18	52.73	1
